# Association between exposure to air pollutants and cardiovascular disease mortality in coastal area of eastern China

**DOI:** 10.3389/fpubh.2025.1543602

**Published:** 2025-06-12

**Authors:** Yiwen Cao, Xiaoyun Zhou, Zaixiang Tang

**Affiliations:** ^1^The Second Affiliated Hospital of Soochow University, School of Public Health, Jiangsu Key Laboratory of Preventive and Translational Medicine for Major Chronic Non-communicable Diseases, MOE Key Laboratory of Geriatric Diseases and Immunology, Suzhou Medical College of Soochow University, Suzhou, China; ^2^Department of Chronic Disease Prevention and Control, Rudong Center for Disease Control and Prevention, Nantong, China

**Keywords:** cardiovascular disease, air pollutants, excess deaths, time-series study, generalized additive model, coastal area

## Abstract

**Background:**

The impact of air pollutants on cardiovascular disease (CVD) mortality remains inadequately studied in Rudong, an eastern coastal area of China. This study aimed to investigate the association between air pollutants and CVD mortality in Rudong.

**Methods:**

Daily number of deaths from CVD, meteorological and air pollutants data in Rudong from 2013 to 2022 were collected. The generalized additive model (GAM) was employed to analyze the relationship between air pollutants and CVD mortality, and stratified analyses were conducted by gender, age, and season.

**Results:**

There was a significant association between air pollutants and CVD mortality. A total of 36,972 deaths from CVD-related deaths were included in the study. We observed that short-term exposure to PM_2.5_, PM_10_, SO_2_, CO, and O_3_ was positively correlated with CVD mortality. Per 10 μg/m^3^ increment in PM_2.5_ (lag05), PM_10_ (lag05), SO_2_ (lag05), CO (lag04), and O_3_ (lag06) (per 1 mg/m^3^ increment in CO), the excess risk of CVD mortality were 1.00% (95%CI:0.37%, 1.64%), 1.05% (95%CI:0.15%, 1.96%), 7.65% (95%CI:4.47%,10.94%), 13.82% (95%CI:4.47%,23.99%), and 1.82% (95%CI:1.02%, 2.62%), respectively. Overall, the estimated impact of air pollutants was greater in the warm season. In addition, susceptibility to air pollution exposure varied across different genders and age groups, with females and those over 65 years old being more sensitive.

**Conclusion:**

Exposure to air pollutants increased the risk of CVD mortality. Furthermore, the health effects of air pollution may be influenced by season, gender, and age. In conclusion, reducing pollutant concentrations to lower levels may provide greater cardiovascular benefits.

## 1 Introduction

In recent years, many studies have shown that air pollution is closely related to the occurrence, development and mortality of CVD (1–4). In China, air pollution remains a critical environmental challenge. Particulate matter (PM) is major component of air pollutants, with fine particles less than 2.5 μm in diameter (PM_2.5_) being particularly concerning due to their small size and large surface area. These particles can adsorb toxic and harmful substances, remain suspended in the atmosphere for extended periods, and be inhaled into the human respiratory system. Once inhaled, PM_2.5_ deposits in the respiratory and cardiovascular systems, leading to varying degrees of health damage, including increased mortality risk. In addition to particulate matter, gaseous pollutants such as sulfur dioxide (SO₂), nitrogen dioxide (NO_2_), carbon monoxide (CO), and ozone (O_3_) also pose serious threats to human health. Gaseous pollutants are more likely to spread through the respiratory tract, stimulating areas such as the lungs, organs, and bronchi, causing irreversible damage to the body. Upon entering the bloodstream, they may induce systemic effects, such as respiratory distress and hypoxia. Then they will affect cardiovascular function and lead to the occurrence of diseases and death.

Currently, CVD remains the leading cause of death worldwide and its burden is increasing. From 1990 to 2019, the global prevalence of CVD increased from 271 million to 523 million cases, while CVD-related deaths rose from 12.1 million to 18.6 million (5). In 2019, CVD accounted for 46.74 and 44.26% of all deaths in rural and urban areas in China (6). According to the “China Cardiovascular Health and Disease Report 2023,” it is estimated that there are 330 million CVD patients in China. With the process of population aging and social urbanization, the incidence rate and prevalence of related diseases are still rising in China.

The pathogenesis of CVD is complex, influenced not only by biological factors but also by social and environmental determinants (7). The Global Burden of Disease Study indicated that in 2019, ambient particulate matter pollution (APMP) was responsible for 14.78% of total CVD deaths, and the age-standardized mortality rate (ASMR) in low- and middle-income regions was 2.3 times higher than in high-income regions (8). Compared to genetic factors or individual behaviors, air pollution could be mitigated at the population level through policy interventions, whereas traditional behavioral interventions (such as smoking cessation) were effective but more difficult to implement (9). Therefore, studying the association between air pollution and CVD mortality holds significant public health value.

Numerous studies have examined the impact of particulate matter on CVD mortality. Research based on the China-PAR project demonstrated that each 10 μg/m^3^ elevation in PM_2.5_ concentration was associated with a 22% heightened risk of CVD mortality (10). Furthermore, gaseous pollutants also exert a significant influence on CVD mortality rates. Large-scale time-series studies conducted in China found that each increment of 10 μg/m^3^ in SO_2_, NO_2_, CO, and O_3_ concentrations corresponded to increases in CVD mortality risk of 0.7, 0.9, 1.12%, and 0.27%, respectively (11–14). Similarly, a major Canadian cohort study confirmed that both O_3_ and NO_2_ contribute to an increased risk of CVD mortality (15). Collectively, these findings indicate that various air pollutants exhibit differential hazardous effects on CVD-related fatalities.

Time series models are widely employed to investigate the acute health effects of air pollution. Among these, the generalized additive model (GAM) is the most commonly used due to its ability to capture both linear and nonlinear relationships between air pollutants and health outcomes. GAMs are particularly valuable because they allow for the incorporation of meteorological factors, long-term trends, and co-pollutants, enabling effective multi-factor adjustments. This capacity to control for confounding variables enhances the accuracy of assessments of the environmental impact on public health. Consequently, GAMs provide a more precise evaluation of how environmental factors affect population health.

Coastal areas, characterized by dense industrial activity, developed transportation networks, and high levels of urbanization, face a challenging air pollution situation. The combination of the sea-land wind system and high humidity climate in these regions tends to facilitate the secondary formation of pollutants, thereby exacerbating the toxicity of particulate matter. Additionally, it is widely recognized that the older adult population is at high risk for CVD. In recent years, the aging process in coastal areas has accelerated significantly, leading to a more pronounced burden of CVD in these areas. It is of great significance to study the relationship between air pollution and CVD mortality in coastal areas. Therefore, this study aims to evaluate the association between air pollutants and CVD mortality in Rudong, a coastal area of eastern China, from 2013 to 2022. Additionally, stratified analyses by age, gender, and season were conducted to identify particularly susceptible subpopulations.

## 2 Materials and methods

### 2.1 Study population

**Figure 1:**
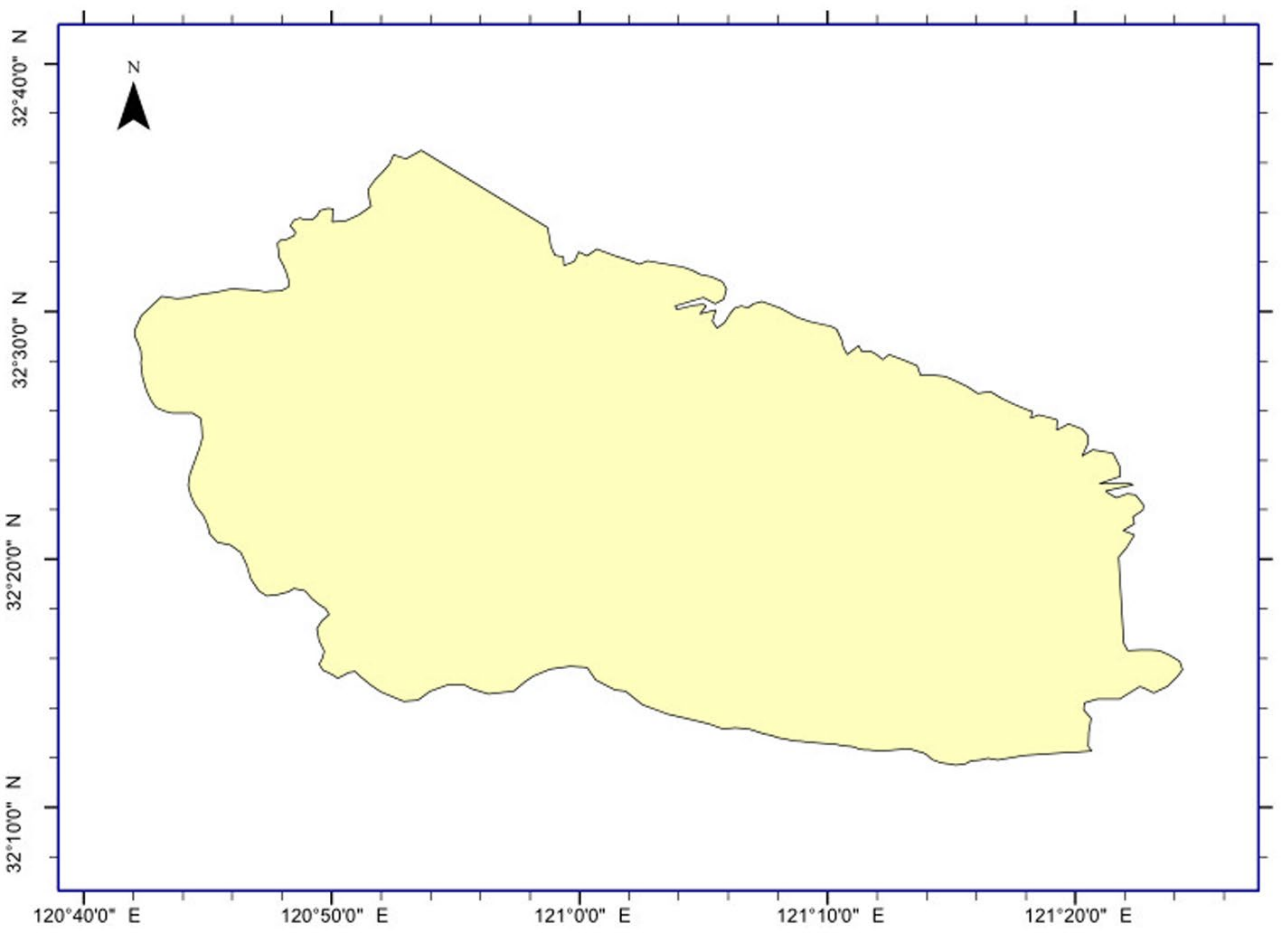
Schematic diagram of the geographical location of Rudong.

Rudong, a coastal city in eastern China with a developed economy and ample medical resources, experiences distinct air pollution patterns due to its subtropical monsoon climate. In recent years, rapid industrial growth has worsened air pollution. At the end of 2022, Rudong had a population of approximately 986,000, of which about 29.0% were aged 65 and above. The older adult are commonly regarded as the primary risk group for CVD. These factors—climatic specificity, unique pollution profiles, and an aging society—render Rudong an epidemiologically significant location for studying the environmental health impacts on CVD burdens (Figure 1).

We obtained daily death data from the Center for Disease Control and Prevention from January 1, 2013 to December 31, 2022. The dataset includes the deceased’s age, gender, date of death, and primary cause of death coded according to the 10th edition of the International Classification of Diseases (ICD-10), where cardiovascular diseases (CVD) are classified as I00–I99.

### 2.2 Meteorological and pollution data

Daily air pollution data were provided by National Tibetan Plateau / Third Pole Environment Data Center (http://data.tpdc.ac.cn) (16–26). The dataset provides raster data on PM_2.5_, PM_10_, SO_2_, NO_2_, CO, and O_3_. We computed the daily average concentration of air pollutants within the study area based on spatial grid data and administrative boundaries. Daily meteorological data, including mean temperature and relative humidity, were obtained from the National Meteorological Science Data Center. These data were matched with air pollution data using spatial averaging methods to ensure consistency across the study region.

### 2.3 Statistical analysis

The main objective of this study is to evaluate the relationship between short-term exposure to air pollutants and CVD mortality. We conducted descriptive analysis on daily CVD deaths, air pollutants, and meteorological variables. Spearman correlation analysis was used to examine the correlation between air pollutants and meteorological variables.

The daily mortality of residents due to CVD is a low-probability event, and its distribution tends to follow a Poisson distribution. Consequently, our study employed a Generalized Additive Model (GAM) to analyze the association between air pollutant exposure and daily CVD mortality among residents while accounting for the confounding effects of other pollutants and meteorological factors. The formula for GAM is as follows:


$$\begin{array}{l} \text{logE}(\mathrm{Yt})={\mathrm{\beta Z}}_{t}+\mathrm{ns}(\text{time},\mathrm{df})+\mathrm{ns}(\text{temp},\mathrm{df})\\ +\mathrm{ns}(\mathrm{rh},\mathrm{df})+\mathrm{DOW}+\alpha \end{array}$$

where E(Yt) represents the estimated number of deaths caused by CVD at day t; *α* represents the intercept of the regression model; $Z_{t}$ denotes the daily average concentration of air pollutants at day t, measured in μg/m^3^; β represents the logarithm of the relative rate of daily CVD mortality associated with a 10 μg/m^3^ increase in pollution concentration ( $Z_{t}$ ) (with CO set at 1 μg/m^3^); DOW , as a binary variable, divided into weekdays and weekends, is introduced into the model as a factor variable, excluding natural fluctuations in daily deaths over the week; ns(time,df) is a natural cubic spline smoothing function that adjusts for death dates (time) based on multi-year and seasonal trends; ns(temp,df) and ns(rh,df) are natural cubic spline smoothing functions for two weather variables ( temperature,relative humidity ); df denotes degrees of freedom. The Akaike Information Criterion (AIC) was used to evaluate the goodness of fit of a model, and the smaller the AIC, the better the goodness of fit of the model. In this model, 8df (per year) was chosen as the time variable, and both temperature and relative humidity were set at 6df .

To assess lagged effects, we constructed both single-day lag models (lag 0 to lag 7, where lag 0 represents same-day exposure and lag 7 represents exposure 7 days prior) and multi-day moving average models (lag 01 to lag 07, where lag 01 represents the average concentration of the current and previous day, and lag 07 represents the 7-day moving average). In addition, we also conducted stratified analysis based on sex (male, female) and age (20 ~ 64 years, 65 ~ years). Seasonal effects were examined by analyzing warm (April–September) and cold (October–March) seasons separately. The results reported the excess risk (ER) and 95% confidence interval (CI) of daily CVD deaths associated with a 10 μg/m^3^ increase in air pollutants (1 mg/m^3^ increase in CO).


ER=[exp(β∗10)−1]∗100%

We selected the lag time corresponding to the maximum ER value for each pollutant, then fitted multi-pollutant models. We verified model robustness by comparing results between single- and multi-pollutant models. Besides, we conducted sensitivity analysis to evaluate the robustness of the results by changing the df (6–8) of time. Models are considered robust if the effect estimated derived from these models did not show significant differences. Lastly, the exposure-response (ER) curve depicting the relationship between CVD mortality and air pollutants was constructed based on GAM.

All statistical analyses were conducted in the R software (version 4.3.1), with the mgcv package for GAM modeling and *ggplot2* for visualization.

## 3 Results

### 3.1 Descriptive results

Table 1 presents descriptive statistics for daily CVD deaths, air pollutants, and meteorological data from January 1, 2013, to December 31, 2022. A total of 36,972 CVD deaths were recorded, with an average daily mortality of 10.13. Of these, 51.9% were female, and 93.5% were over 65 years old. The daily average concentrations of PM_10_, PM_2.5_, NO_2_, SO_2_, CO, and O_3_ were 67.15 μg/m^3^, 40.45 μg/m^3^, 25.71 μg/m^3^, 15.45 μg/m^3^, 0.73 mg/m^3^, and 105.10 μg/m^3^, respectively. Throughout the study, the average concentrations of NO_2_, SO_2_, and CO remained below primary air quality standards, while PM_10_, PM_2.5_, and O_3_ exceeded primary standards but stayed below secondary standards. The daily average temperature was 16.3°C, with a relative humidity of 77.4%.

**Table 1:** Descriptive statistics of daily CVD deaths, air pollutants and meteorological data from 2013 to 2022.

Variables	Mean	Min	P_25_	Median	P_75_	Max
Total death	10.13	0	7	10	13	51
<65 years old	0.66	0	0	1	1	5
≥65 years old	9.47	0	7	9	12	49
Male	4.88	0	3	5	6	26
Female	5.25	0	3	5	7	27
PM_10_ (μg/m^3^)	67.15	11.73	40.35	58.19	84.79	331.82
PM_2.5_(μg/m^3^)	40.45	5.59	22.30	33.88	51.03	249.97
NO_2_ (μg/m^3^)	25.71	7.06	16.71	21.91	30.83	103.86
SO_2_ (μg/m^3^)	15.45	5.05	8.57	13.42	18.57	68.44
CO (mg/m^3^)	0.73	0.36	0.56	0.67	0.84	6.37
O_3_ (μg/m^3^)	105.10	18.21	77.28	99.93	126.98	246.85
Temp (°C)	16.3	−7.6	8.4	16.7	23.8	34.7
RH (%)	77.4	33	70	79	86	100

**Figure 2:**
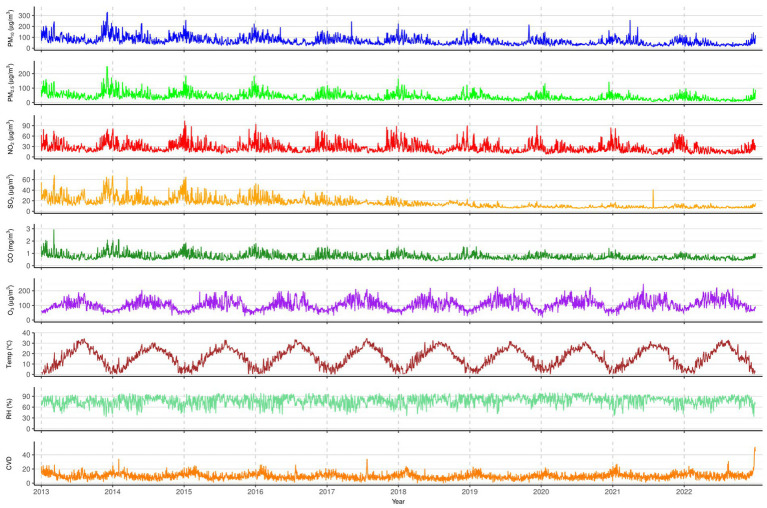
The distribution of air pollutant concentrations, and daily CVD deaths from 2013 to 2022.

Figure 2 illustrates the time series of daily air pollutants and CVD deaths from 2013 to 2022. CVD deaths exhibited clear seasonality, peaking in winter and dipping in summer, with a consistent annual trend. Air pollutants (except O_3_) also followed this seasonal pattern. In contrast, O_3_ concentrations were higher in summer and lower in winter.

### 3.2 Correlation analysis

**Figure 3:**
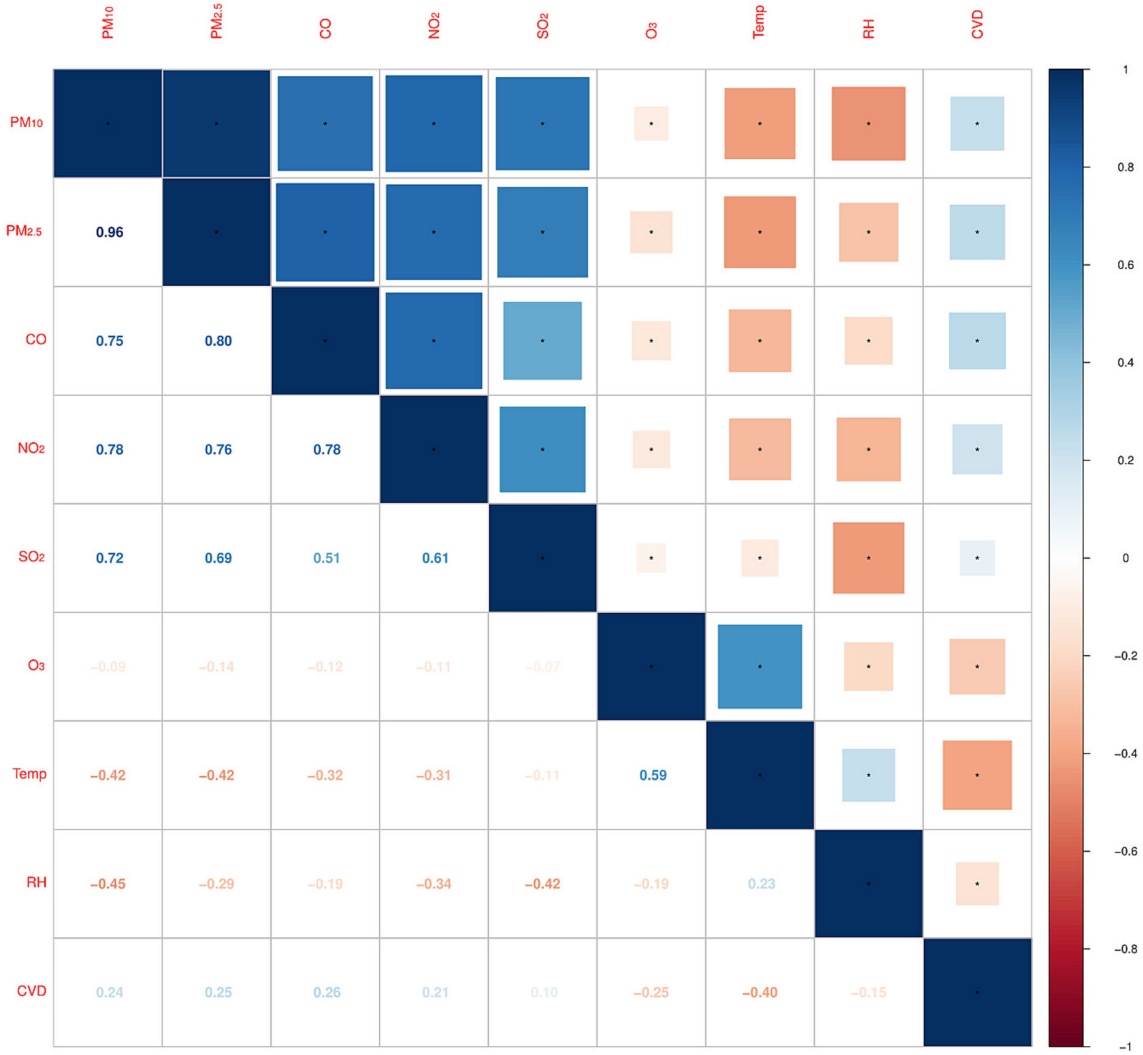
Spearman correlation between daily meteorological factors and air pollutants. (*Correlation is significant at the 0.01 level).

Pearson correlation analysis revealed that O_3_ was negatively correlated with PM_10_, PM_2.5_, NO_2_, SO_2_, and CO, and positively correlated with temperature. Excluding O_3_, the other five pollutants were positively correlated with each other, with the strongest correlation between PM_2.5_ and PM_10_ (*r* = 0.96, *p* < 0.01). Temperature was negatively correlated with all pollutants except O_3_, while relative humidity was negatively correlated with all pollutants and positively correlated with temperature. Additionally, daily CVD deaths were positively correlated with PM_10_, PM_2.5_, NO_2_, SO_2_, and CO concentrations, and negatively correlated with O_3_, temperature, and relative humidity, with all correlations being statistically significant (Figure 3).

### 3.3 Regressive results

Table 2 shows the lag-response of the association between air pollutants and CVD mortality. In addition to NO_2_, all air pollutants had a significant positive correlation with CVD mortality. For single-day lags, the greatest effect of air pollutants on CVD mortality was found at lag3. For 10 μg/m^3^ increase in PM_10_, SO_2_ and O_3_, the ER of CVD mortality was 0.45% (95%CI:0.08%, 0.83%), 3.26% (95%CI: 1.45%, 5.10%), 0.94% (95%CI: 0.50%, 1.39%). For multi-day lags, we observed that the estimated impact of all cumulative exposures was greater than that of single-day lags. Generally, the estimated ER in CVD mortality associated with a 10 μg/m^3^ increment in pollutant concentrations mostly reached a maximum at lag 05, up to 1.00% (95%CI: 0.37%,1.64%) for PM10, 1.05% (95%CI:0.15%, 1.96%) for PM2.5, 7.65% (95%CI: 4.47%, 10.94%) for SO2,1.82% (95%CI: 1.02%, 2.62%) for O3, respectively. The largest estimate for CO was found at lag 04, the ER was 13.82% (95%CI: 4.47%, 23.99%).

**Table 2:** Excess relative risk (ER) and 95% confidence interval (CI) of CVD mortality associated with a 10 μg/m^3^ increment in air pollutant concentration (1 mg/m^3^ in CO), 2013–2022.

Lag days	PM_10_	PM_2.5_	NO_2_	SO_2_	CO	O_3_
lag0	0.28(−0.11,0.67)	0.43(−0.10,0.96)	0.73(−0.27,1.74)	**2.90(0.88,4.95)**	**6.75(1.31,12.48)**	0.55(−0.01,1.11)
lag1	0.31(−0.08,0.70)	0.28(−0.26,0.81)	0.29(−0.70,1.29)	**2.89(0.95,4.86)**	2.48(−2.72,7.96)	**0.68(0.19,1.17)**
lag2	**0.45(0.08,0.82)**	0.39(−0.13,0.91)	0.03(−0.90,0.98)	**3.13(1.29,5.01)**	4.26(−0.87,9.66)	**0.76(0.30,1.21)**
lag3	**0.45(0.08,0.83)**	0.44(−0.08,0.95)	0.72(−0.19,1.64)	**3.26(1.45,5.10)**	**6.31(1.17,11.71)**	**0.94(0.50,1.39)**
lag4	0.32(−0.04,0.69)	0.46(−0.05,0.97)	0.33(−0.57,1.23)	**2.18(0.40,4.00)**	2.39(−2.56,7.60)	**0.57(0.13,1.01)**
lag5	0.24(−0.12,0.61)	0.12(−0.39,0.63)	−0.21(−1.11,0.69)	1.26(−0.52,3.07)	−1.04(−5.85,4.00)	0.28(−0.16,0.72)
lag6	−0.21(−0.58,0.15)	−0.43(−0.94,0.08)	**−1.51(−2.40,-0.61)**	−0.91(−2.67,0.87)	**−6.86(−11.42,-2.08)**	0.17(−0.27,0.60)
lag7	**−0.52(−0.89,-0.15)**	**−0.66(−1.17,-0.15)**	**−1.46(−2.35,-0.56)**	**−2.37(−4.10,-0.61)**	**−8.16(−12.65,-3.44)**	−0.17(−0.61,0.26)
lag01	0.39(−0.06,0.83)	0.46(−0.15,1.07)	0.76(−0.44,1.98)	**3.90(1.63,6.23)**	**6.74(0.28,13.62)**	**0.92(0.29,1.56)**
lag02	**0.59(0.09,1.09)**	0.61(−0.07,1.30)	0.72(−0.63,2.10)	**5.15(2.63,7.72)**	**8.92(1.46,16.94)**	**1.25(0.57,1.92)**
lag03	**0.78(0.24,1.33)**	**0.81(0.05,1.58)**	1.19(−0.31,2.71)	**6.51(3.75,9.34)**	**12.69(4.18,21.90)**	**1.65(0.94,2.37)**
lag04	**0.91(0.32,1.51)**	**1.02(0.19,1.86)**	1.34(−0.27,2.98)	**7.26(4.29,10.32)**	**13.82(4.47,23.99)**	**1.80(1.05,2.56)**
lag05	**1.00(0.37,1.64)**	**1.05(0.15,1.96)**	1.19(−0.52,2.93)	**7.65(4.47,10.94)**	**12.98(3.01,23.93)**	**1.82(1.02,2.62)**
lag06	**0.88(0.21,1.57)**	0.82(−0.15,1.79)	0.31(−1.49,2.14)	**7.11(3.73,10.60)**	8.61(−1.68,19.97)	**1.81(0.97,2.65)**
lag07	0.63(−0.09,1.36)	0.49(−0.54,1.53)	−0.49(−2.38,1.45)	**5.95(2.39,9.63)**	3.71(−6.78,15.38)	**1.65(0.78,2.54)**

Bold values indicate statistical significance (*p* < 0.05).

**Figure 4:**
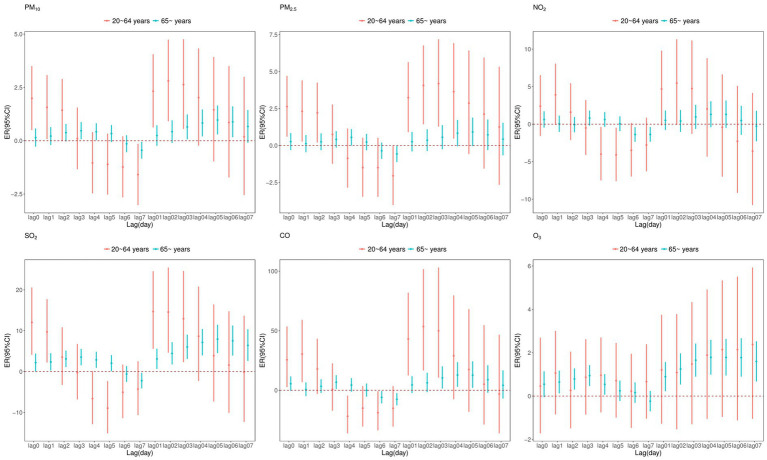
Excess relative risk (ER) and 95% confidence interval (CI) of CVD mortality associated with air pollutants (per 10 μg/m^3^ increase and 1 mg/m^3^ increase in CO) at various lags stratified by age in single pollutant model.

Figure 4 shows ER with 95%CI of CVD mortality associated with air pollutants at different lag times stratified by age. For those aged 20–64 years, PM_10_, PM_2.5_, SO_2_, and CO were significantly positively correlated with CVD mortality. For those over 65 years old, all pollutants except PM_2.5_ and NO_2_ had a significant impact on CVD mortality. In our study, during a lag period of 0 to 3 days, the adverse effects of air pollutants (excluding O_3_) were marginally more pronounced in individuals under the age of 65 compared to those over 65. After a lag period exceeding 3 days, the impact of air pollutants on individuals aged 65 and above was significantly greater.

**Figure 5:**
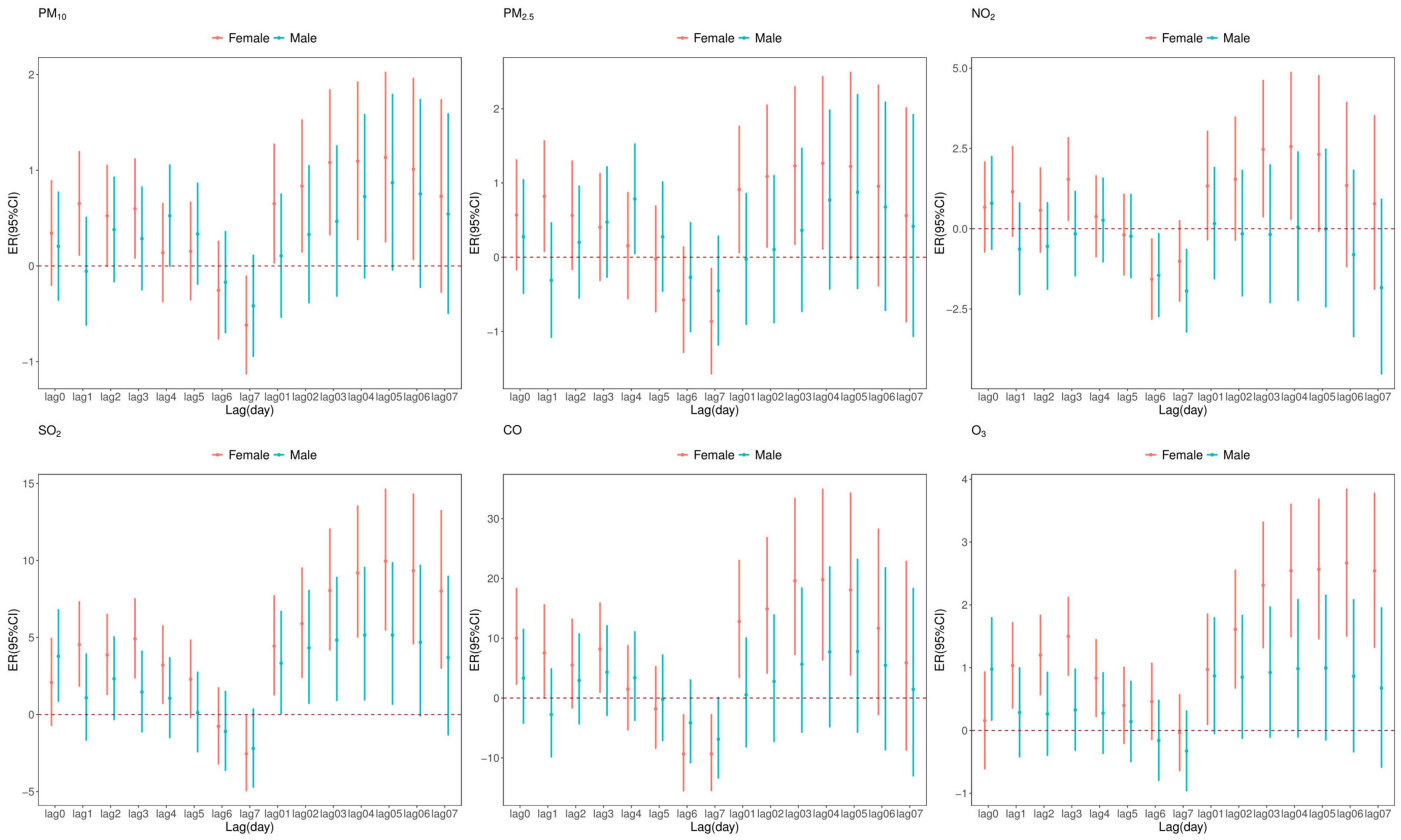
Excess relative risk (ER) and 95% confidence interval (CI) of CVD mortality associated with air pollutants (per 10 μg/m^3^ increase and 1 mg/m^3^ increase in CO) at various lags stratified by sex in single pollutant model.

Figure 5 presents a gender-stratified analysis of the impact of air pollutants on CVD mortality. The results show that exposure to all pollutants increased the risk of CVD mortality in female, whereas for male, only exposure to SO_2_, CO and O_3_ was associated with an increased risk. Furthermore, the effect of air pollution on CVD mortality was more pronounced in female.

**Figure 6:**
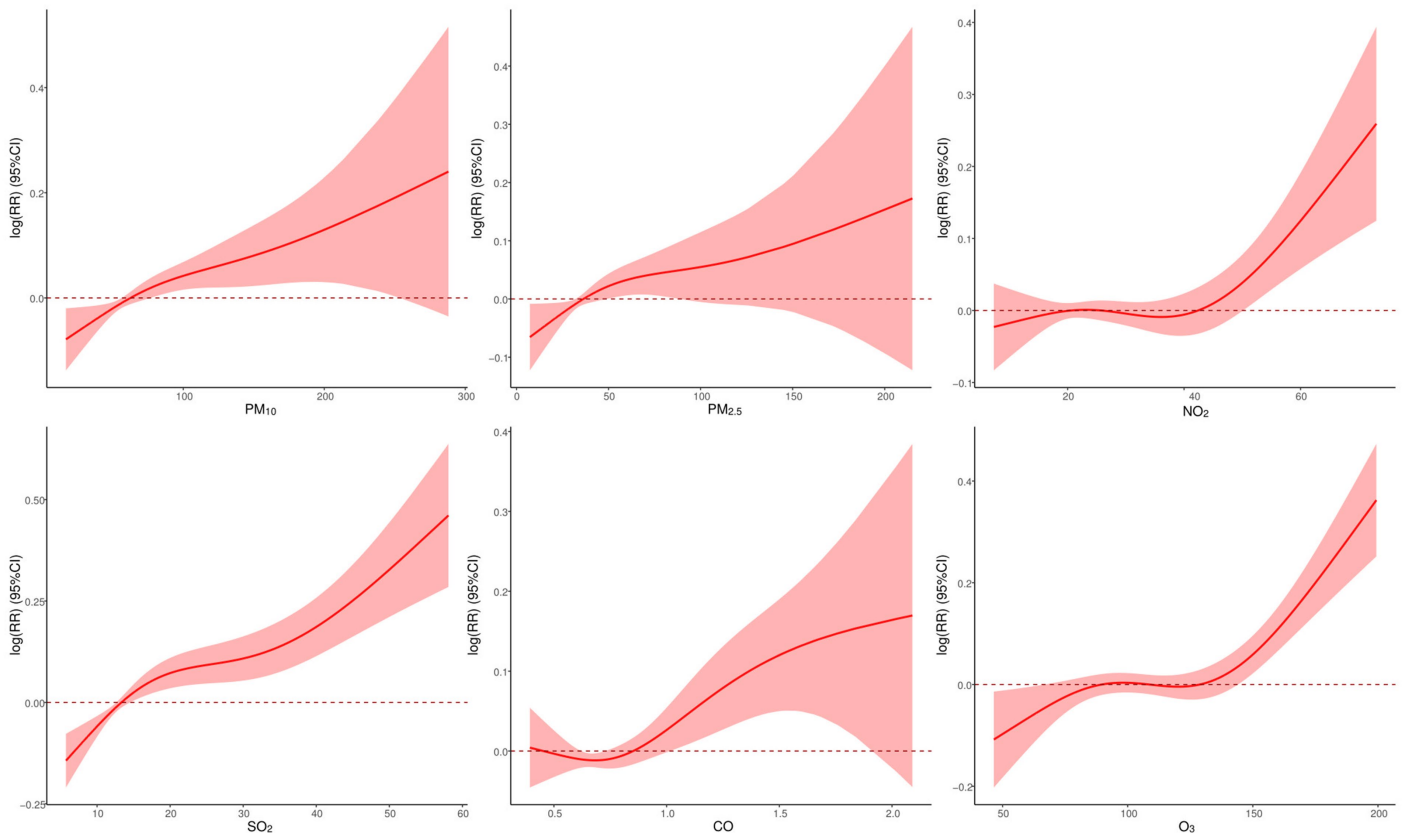
Exposure-response curves between air pollutants and CVD mortality.

Figure 6 displays exposure-response (E-R) curves between air pollutants and CVD mortality. Overall, these curves exhibited a positive nonlinear trend, indicating that higher exposure to air pollution might be associated with a non-proportional increase in CVD mortality. The E-R curves for PM_2.5_, PM_10_, and CO initially exhibited a steady increase at low concentrations, but once they reached a certain level, the curves no longer possessed statistical significance. Conversely, the E-R relationship curves for NO_2_, SO_2_, and O_3_ demonstrated a gradual increase in the initial stages, followed by a rapid growth without threshold once they attained a specific concentration.

**Figure 7:**
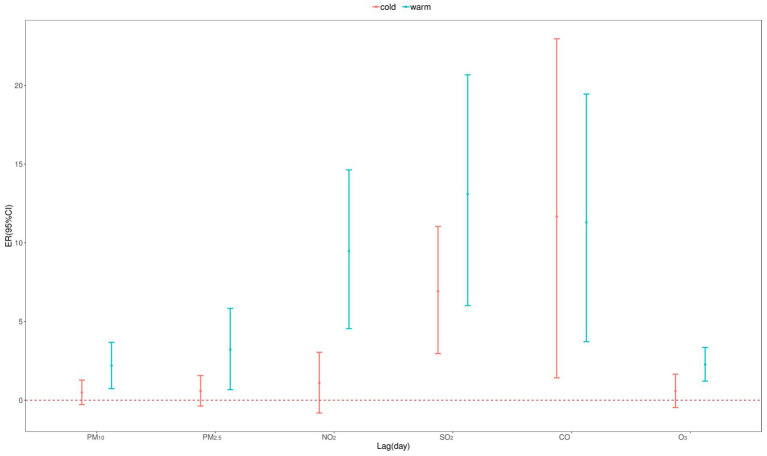
Excess relative risk (ER) and 95% confidence interval (CI) of CVD mortality with an increase of 10 μg/m^3^ in air pollutants (1 mg/m^3^ in CO) by season in single pollutant model.

According to the seasonal stratification analysis (Figure 7, Tables 3, 4), short-term exposure to all pollutants during the warm season was significantly positively correlated with CVD mortality, while only SO_2_ and CO had a statistically significant adverse effect on CVD mortality during the cold season. The impact of air pollutants on CVD was greater in warm seasons than in cold seasons.

**Table 3:** ERs (95% CIs) of CVD mortality associated with a 10 μg/m^3^ increment in air pollutant concentration (1 mg/m^3^ in CO) in cold season.

Lag days	PM_10_	PM_2.5_	NO_2_	SO_2_	CO	O_3_
lag0	0.15(−0.30,0.59)	0.27(−0.31,0.86)	0.97(−0.14,2.09)	**3.42(1.07,5.82)**	4.80(−1.34,11.32)	0.59(−0.47,1.65)
lag1	0.10(−0.35,0.55)	0.12(−0.47,0.71)	0.25(−0.86,1.37)	2.20(−0.07,4.51)	2.32(−3.68,8.70)	−0.11(−1.09,0.88)
lag2	0.23(−0.21,0.67)	0.22(−0.36,0.80)	−0.29(−1.34,0.77)	1.73(−0.46,3.97)	2.97(−2.93,9.24)	−0.05(−1.01,0.92)
lag3	0.20(−0.23,0.64)	0.20(−0.37,0.78)	0.47(−0.56,1.51)	**2.57(0.39,4.79)**	5.55(−0.42,11.88)	−0.93(−1.86,0.02)
lag4	0.10(−0.33,0.53)	0.16(−0.41,0.73)	0.00(−1.01,1.03)	1.29(−0.84,3.48)	−0.69(−6.35,5.30)	**−1.13(−2.05,-0.19)**
lag5	0.04(−0.39,0.47)	−0.13(−0.70,0.44)	−0.29(−1.30,0.73)	0.98(−1.16,3.17)	−1.78(−7.36,4.14)	−0.73(−1.65,0.20)
lag6	−0.19(−0.62,0.24)	−0.41(−0.97,0.16)	**−1.28(−2.28,-0.26)**	−1.05(−3.16,1.12)	**−6.08(−11.44,-0.40)**	−0.52(−1.44,0.41)
lag7	−0.54(−0.97,-0.11)	−0.65(−1.21,-0.08)	**−1.03(−2.03,-0.02)**	**−2.31(−4.40,-0.18)**	**−6.39(−11.72,-0.74)**	**−1.14(−2.06,-0.21)**
lag01	0.16(−0.35,0.68)	0.26(−0.42,0.94)	0.94(−0.41,2.31)	**3.78(1.13,6.51)**	5.49(−1.98,13.53)	0.32(−0.93,1.60)
lag02	0.29(−0.28,0.87)	0.37(−0.40,1.15)	0.71(−0.85,2.29)	**4.39(1.42,7.45)**	7.45(−1.32,17.00)	0.24(−1.18,1.68)
lag03	0.41(−0.24,1.06)	0.49(−0.38,1.36)	1.08(−0.67,2.86)	**5.71(2.39,9.15)**	**11.67(1.41,22.96)**	−0.43(−1.99,1.16)
lag04	0.47(−0.24,1.19)	0.59(−0.37,1.56)	1.10(−0.82,3.04)	**6.34(2.69,10.11)**	**11.40(0.06,24.02)**	−1.16(−2.85,0.56)
lag05	0.49(−0.28,1.28)	0.50(−0.56,1.57)	0.93(−1.12,3.02)	**6.92(2.96,11.04)**	10.56(−1.74,24.41)	−1.57(−3.38,0.28)
lag06	0.39(−0.45,1.24)	0.25(−0.90,1.41)	0.09(−2.09,2.32)	**6.56(2.28,11.02)**	6.31(−6.57,20.98)	−1.86(−3.79,0.11)
lag07	0.11(−0.80,1.02)	−0.13(−1.37,1.12)	−0.57(−2.89,1.81)	**5.54(0.95,10.33)**	1.76(−11.59,17.14)	**−2.57(−4.62,-0.48)**

Bold values indicate statistical significance (*p* < 0.05).

**Table 4:** ERs (95% CIs) of CVD mortality associated with a 10 μg/m^3^ increment in air pollutant concentration (1 mg/m^3^ in CO) in warm season.

Lag days	PM_10_	PM_2.5_	NO_2_	SO_2_	CO	O_3_
lag0	0.59(−0.47,1.65)	0.09(−1.50,1.71)	−0.10(−3.08,2.96)	−2.10(−6.65,2.67)	3.55(−4.46,12.24)	0.00(−0.76,0.76)
lag1	−0.11(−1.09,0.88)	0.27(−1.28,1.84)	1.39(−1.24,4.10)	3.82(−0.38,8.19)	−1.56(−9.58,7.17)	**0.62(0.02,1.23)**
lag2	−0.05(−1.01,0.92)	0.74(−0.71,2.21)	**3.37(0.92,5.89)**	**6.74(2.85,10.78)**	3.63(−3.88,11.74)	**0.71(0.17,1.26)**
lag3	−0.93(−1.86,0.02)	**1.45(0.04,2.89)**	**4.00(1.60,6.46)**	**6.04(2.25,9.97)**	3.45(−3.88,11.33)	**1.22(0.69,1.76)**
lag4	**−1.13(−2.05,-0.19)**	**2.33(0.92,3.77)**	**4.23(1.84,6.67)**	**6.92(3.12,10.87)**	**11.31(3.71,19.45)**	**0.95(0.42,1.48)**
lag5	−0.73(−1.65,0.20)	1.33(−0.08,2.76)	1.54(−0.80,3.94)	2.74(−0.95,6.56)	2.27(−5.51,10.69)	0.48(−0.05,1.00)
lag6	−0.52(−1.44,0.41)	−1.13(−2.53,0.28)	−1.48(−3.77,0.86)	−0.13(−3.73,3.61)	−5.43(−13.35,3.20)	0.16(−0.36,0.69)
lag7	**−1.14(−2.06,-0.21)**	−1.27(−2.67,0.14)	−3.06(−5.32,-0.74)	−2.71(−6.27,0.98)	−9.80(−17.59,-1.27)	−0.01(−0.53,0.52)
lag01	0.32(−0.93,1.60)	0.24(−1.58,2.10)	1.15(−2.32,4.75)	1.72(−3.48,7.19)	1.79(−8.74,13.55)	0.61(−0.23,1.47)
lag02	0.24(−1.18,1.68)	0.68(−1.32,2.72)	3.72(−0.13,7.72)	**6.20(0.53,12.19)**	4.96(−7.48,19.07)	**1.04(0.17,1.91)**
lag03	−0.43(−1.99,1.16)	1.42(−0.76,3.65)	**6.40(2.17,10.80)**	**9.23(3.09,15.74)**	7.25(−6.60,23.17)	**1.72(0.82,2.63)**
lag04	−1.16(−2.85,0.56)	**2.57(0.21,4.99)**	**8.80(4.20,13.60)**	**12.19(5.53,19.27)**	15.56(−0.39,34.06)	**2.13(1.18,3.08)**
lag05	−1.57(−3.38,0.28)	**3.22(0.67,5.83)**	**9.47(4.55,14.63)**	**13.10(6.01,20.67)**	16.70(−0.56,36.95)	**2.27(1.27,3.29)**
lag06	−1.86(−3.79,0.11)	2.58(−0.15,5.39)	**8.09(2.91,13.53)**	**12.42(4.94,20.43)**	12.86(−5.10,34.23)	**2.27(1.21,3.35)**
lag07	**−2.57(−4.62,-0.48)**	1.83(−1.09,4.83)	**5.48(0.10,11.14)**	**9.68(1.99,17.95)**	5.85(−12.26,27.70)	**2.23(1.10,3.36)**

Bold values indicate statistical significance (*p* < 0.05).

### 3.4 Sensitivity analysis results

**Figure 8:**
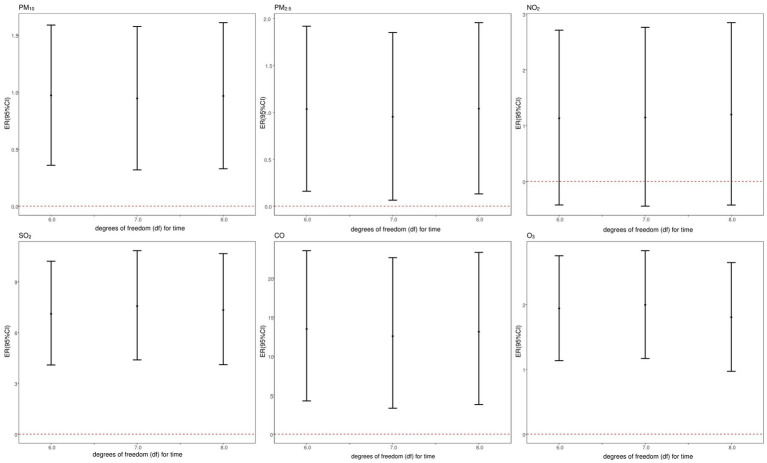
Sensitivity analysis of association between CVD mortality and air pollutants: controlling for different degrees of freedom (df) for time.

After further adjusting the calendar time df ( 6−8df per year) (as shown in the Figure 8), the estimated impact of air pollutants (excluding NO_2_) on CVD mortality slightly changed, but the effect remained significant.

The results of the multi-pollutant model are shown in Table 5. The results indicated that the estimated effect of pollutants on CVD mortality changed when additional pollutants were incorporated into the model. After accounting for potential confounding effects from co-pollutants, the effect of SO_2_ and O_3_ remained significant, with a slight enhancement in the estimated effect of SO_2_ and a minor reduction in the estimated effect of O_3_.

**Table 5:** ERs (95%CI) for the air pollutants per 10 mg/m^3^ increase in the concentration for CVD mortality (multi-pollutant model).

Pollutant	Models	ER (95%CI)
PM_10_		**1.00(0.37, 1.64)**
	+PM_2.5_	**2.00(0.33, 3.70)**
	+NO_2_	**1.21(0.34, 2.08)**
	+SO_2_	−0.06(−0.92, 0.80)
	+CO	0.65(−0.29, 1.60)
	+O_3_	0.54(−0.14, 1.22)
	+PM_2.5_ + NO_2_ + SO_2_ + CO + O_3_	0.95(−0.97, 2.91)
PM_2.5_		**1.05(0.15, 1.96)**
	+PM_10_	−1.57(−3.86, 0.78)
	+NO_2_	1.01(−0.14, 2.16)
	+SO_2_	−0.29(−1.40, −0.83)
	+CO	0.12(−1.27, 1.54)
	+O_3_	0.46(−0.48, 1.42)
	+PM_10_ + NO_2_ + SO_2_ + CO + O_3_	−1.62(−4.19, 1.01)
SO_2_		**7.65(4.47, 10.94)**
	+PM_10_	**7.60(3.24, 12.14)**
	+PM_2.5_	**8.00(4.02, 12.14)**
	+NO_2_	**11.19(6.62, 15.96)**
	+CO	**7.97(3.60, 12.52)**
	+O_3_	**5.72(2.41, 9.15)**
	+PM_10_ + PM_2.5_ + NO_2_ + CO + O_3_	**8.42(3.36, 13.72)**
CO		**13.82(4.47, 23.99)**
	+PM_10_	5.86(−6.86, 20.33)
	+PM_2.5_	11.97(−2.03, 27.99)
	+NO_2_	**18.22(4.09, 34.27)**
	+SO_2_	−2.25(−13.01, 9.83)
	+O_3_	8.16(−1.05, 18.23)
	+PM_10_ + PM_2.5_ + NO_2_ + SO_2_ + O_3_	10.23(−6.41, 29.84)
O_3_		**1.82(1.02, 2.62)**
	+PM_10_	**1.61(0.77, 2.46)**
	+PM_2.5_	**1.71(0.88, 2.55)**
	+NO_2_	**1.79(0.98, 2.61)**
	+SO_2_	**1.42(0.59, 2.25)**
	+CO	**1.65(0.84, 2.48)**
	+PM_10_ + PM_2.5_ + NO_2_ + SO_2_ + CO	**1.43(0.59, 2.28)**

Bold values indicate statistical significance (*p* < 0.05).

## 4 Discussion

Overall, from January 1, 2013 to December 31, 2022, there were a total of 36,972 CVD deaths in Rudong. We found that air pollutants could increase the risk of CVD mortality, and gaseous pollutants had a greater impact on CVD mortality than particulate matter. For per 10 μg/m^3^ increase of PM_10_ (lag 05), PM_2.5_ (lag 05), SO_2_ (lag 05), CO (lag 04), and O_3_ (lag 05) (1 mg/m^3^ increase of CO), the risk of CVD mortality increased by 1.00, 1.05, 7.65, 13.82, and 1.82%, respectively. The associations between air pollutants and CVD mortality varied by age, gender, and season.

Many studies have investigated the relationship between air pollutants and CVD mortality, yielding mixed results. For instance, a study conducted in Hefei, China, revealed that for every 10 μg/m^3^ increase in SO_2_ and NO_2_ concentrations, the risk of CVD mortality increased by 2.53% (95% CI: 0.86%, 4.21%) and 1.59% (95% CI: 0.15%, 3.04%), respectively (22). A large-scale time-series analysis based on cause-of-death data from 272 cities in China demonstrated that a 10 μg/m^3^ increase in PM_2.5_, SO_2_, NO_2_, and O_3_ concentrations corresponded to increases in cardiovascular mortality risk of 0.25, 0.70, 0.90, and 0.27%, respectively (11–14). These findings collectively indicate a significant correlation between air pollutants and CVD mortality. Furthermore, a global time-series study covering 652 cities showed that for every 10 μg/m^3^ increase in PM_10_ and PM_2.5_ concentrations, CVD mortality rose by 0.36% (95% CI: 0.30%, 0.43%) and 0.55% (95% CI: 0.45%, 0.66%), respectively (23). In comparison, our study estimates a relatively larger effect size. Such discrepancies may be attributed to regional variations in pollutant levels, urban development, chemical composition, and demographic characteristics.

Exposure to ambient air pollutants demonstrated a time-lagged association with CVD mortality. In our study, the single-day lag model showed that PM_10_, SO_2_, and O_3_ had the highest risk at lag 3, while the cumulative lag model indicated peak risks for PM_2.5_, PM_10_, and SO_2_ at lag 05. Dabass et al. conducted a study on air pollution in America, from 1999 to 2011, and found that exposure to PM_2.5_ had the greatest impact on ischemic heart disease and peripheral vascular disease mortality at lag5 (24). A study in Changzhou, China, found that a 0-5 days lag in PM_10_, SO_2_, and NO_2_ concentrations, per interquartile increase, raised ischemic stroke mortality risks by 0.268, 0.34, and 0.263%, respectively (25). A study in Shenzhen showed that both a single-day lag of 4 days and an average lag of 0–4 days presented the highest risks for particulate matter and CVD mortality (26). The differences across various studies may be due to many factors: (1) Variations in the level of air pollutants, composition, and sources may contribute to inconsistent findings. (2) Differences in study designs and analytical approaches could influence results. (3) Variations in population structure, lifestyle, and underlying health conditions might lead to different outcomes.

After incorporating additional pollutants, the effects of air pollutants on CVD mortality were altered. The combination of SO_2_ with other pollutants was associated with an increased risk of CVD mortality, indicating that interactions among pollutants may amplify their adverse effects. In contrast, after adjusting for co-pollutants, the effect of O_3_ on CVD mortality was attenuated, potentially due to the opposing trends between O_3_ and other pollutants. This suggests that pollutant interactions might mitigate the impact on CVD mortality in certain cases.

Our study also identified gender-specific risks. The impact of air pollutants on CVD mortality was more pronounced in female, which was consistent with some previous studies. A time-series study conducted in 16 cities in China found that females have a higher risk of CVD mortality (23), which is consistent with a study in Canada (27). This suggests that females may be more sensitive to air pollution, potentially due to higher prevalence of CVD risk factors, such as hypertension, atrial fibrillation, and diabetes. Additionally, anatomical differences, such as shorter airways, may make females more sensitive to pollutants (28, 29). However, gender differences in air pollution-related CVD mortality remain inconsistent. For example, a study in Rome found PM_2.5_-related CVD mortality risks in males (30), while a study in Shenzhen, China, reported greater risks in males for SO_2_, NO_2_, and PM_10_ (26). A U.S. cohort study showed that each 10 μg/m^3^ increase in PM_2.5_ exposure increased CVD mortality by 10%, with no gender differences (31). These discrepancies may result from variations in population gender ratios, lifestyle habits, and individual characteristics in different regions.

Age stratification analysis revealed that particulate matter had a more substantial impact on CVD mortality in those aged 20–64 years, while gaseous pollutants (SO_2_, O_3_) were associated with a significantly higher risk of mortality in those over 65 years of age. Generally, the older adult are considered more vulnerable to air pollution due to weakened physiological resilience and higher prevalence of chronic diseases. A study in Shenzhen found that every increase of one IQR in SO_2_, NO_2_, and PM_10_, the CVD mortality risk in the older adult (65 ~ years) increased by 4.66, 7.56, and 1.70% (26). Similarly, a study in Chile showed that, compared to younger individuals (<65 years), the CVD mortality risk associated with PM_10_ was more than twice as high in the older adult (32). However, an Iranian study found that individuals <65 years had a higher CVD mortality risk following exposure to air pollution (33). In China, studies conducted in eastern and northeastern regions demonstrated that air pollution disproportionately affected those aged 65 years and older (34, 35). Conversely, a U.S. study reported no significant age-related differences in the effect of PM_2.5_ exposure on CVD mortality risk (31). The differences in these study results might be attributed to variations in air pollutant concentrations, composition, and sources across different regions, and the specific reasons require further investigation.

We conducted a stratified analysis of the seasonal effects on CVD mortality. The results showed that the association between air pollutants and CVD mortality was stronger in warm season. This is consistent with several previous research findings. A study on public health and air pollution in Asia showed that air pollution posed a greater health risk, particularly for cardiorespiratory mortality, during warm seasons (36). Another study in Nanjing found that O_3_ exposure during the warm season had a higher impact on CVD mortality (ER: 0.99%, 95% CI: 0.58%, 1.39%)compared to the cold season (ER: 0.96%, 95% CI: 0.37%, 1.56%). Similarly, a Canadian study found that there was no significant association between O_3_ and CVD mortality during the cold season, but during the warm season, a 10 ppb increase in O_3_ was associated with a 0.7% (95% CI: 0.2%, 1.1%) increase in circulatory system mortality (27). However, our study found that, due to the influence of coal burning and limited wind dispersion, the concentrations of all air pollutants, except O_3_, were higher in the cold season and lower in the warm season. This pattern seemed to contradict the results observed in the seasonal stratification analysis. We hypothesize that the differing impacts of air pollutants on CVD mortality across seasons may be attributed to the following factors: (1) Higher humidity and temperatures in warm seasons may influence pollutant dispersion, thereby increasing their impact on CVD mortality. (2) Residents’ activities vary across different seasons. In the region we selected for our study, agriculture and fisheries are well-developed, people tend to work outdoors more during the warm season, increasing their exposure to air pollutants. In contrast, during the cold season, people spend more time indoors, reducing their exposure to outdoor air pollution. This seasonal variation in exposure may be related to the seasonal differences in the impact of air pollution on CVD mortality risk. Understanding these factors can help develop more targeted interventions to reduce the health impacts of air pollution.

A GBD report indicated that between 1990 and 2019, air pollution was one of the leading contributors to the global disease burden (37). According to the report, air pollution was responsible for 12.2 and 11.3% of all deaths among males and females, respectively. It revealed that air quality standards established by the World Health Organization (WHO), Europe, and the United States were stricter compared to those in China. Reducing air pollutant concentrations to levels recommended by the WHO or lower could potentially prevent more deaths. Consequently, tightening China’s air quality standards may yield greater health benefits.

We employed GAM to analyse the relationship between air pollutants and CVD mortality. In addition, our study selected an eastern coastal area of China, which has unique climatic conditions and population characteristics, so this study is of great significance for improving the burden of CVD in coastal areas. This research is an ecological study, examining the relationship between environmental factors and CVD mortality at the population level. However, it lacks individual data. Additionally, the data in this study has certain regional characteristics. Extrapolation of the research results should be cautious. Future research should adopt other methodologies to further clarify the impact of air pollution on CVD mortality.

## 5 Conclusion

Our study demonstrated that increased levels of air pollutants are associated with a higher risk of death from CVD. The health effects of air pollutants on the cardiovascular system exhibited seasonal variations, with a greater mortality risk during warm seasons compared to cold seasons. Furthermore, the risk of CVD-related death varied across different age and gender groups upon exposure to air pollution. Relevant authorities should enhance their focus on environmental factors, establish early warning systems, and promote public awareness of the health impacts of air pollution. Stricter air quality standards in China could potentially lead to significant improvements in cardiovascular health. These measures hold substantial public health implications for reducing CVD mortality risk.

## Data Availability

The data analyzed in this study is subject to the following licenses/restrictions: the raw data supporting the conclusions of this article will be made available by the authors, without undue reservation. Requests to access these datasets should be directed to caoeven2021@163.com.
